# Prevention and Reduction of Care Against Someone’s Will in Cognitively Impaired People at Home: A Feasibility Study

**DOI:** 10.1093/geroni/igaa057.2302

**Published:** 2020-12-16

**Authors:** Angela Mengelers, Michel Bleijlevens, Hilde Verbeek, Vincent Moermans, Elizabeth Capezuti, Jan Hamers

**Affiliations:** 1 Maastricht University, Maastricht, Limburg, Netherlands; 2 Maastricht University, Riemst, Limburg, Belgium; 3 Hunter College of CUNY, New York, New York, United States

## Abstract

Sometimes care is provided to a cognitively impaired person against the person’s will, referred to as involuntary treatment. An intervention (PRITAH) was developed to prevent and reduce involuntary treatment comprising 4 components: client-centered care policy, workshops, coaching on the job by a specialized nurse and the use of alternative interventions. A feasibility study was conducted including 30 professional caregivers. Feasibility was assessed by attendance lists (reach), a logbook (dose delivered and fidelity), evaluation questionnaires and focus group interviews (dose received, satisfaction & barriers). The workshops and coach were positively evaluated and the average attendance rate was 73%. Participants gained more awareness and knowledge and received practical tips and advice to prevent involuntary treatment. Implementation of the intervention was feasible with minor deviations from protocol. Recommendations for improvement included more emphasis on involvement of family caregivers and general practitioners and development of an extensive guideline to comply with the policy. Part of a symposium sponsored by Systems Research in Long-Term Care Interest Group.

